# Experimental Hyperglycemia Induces an Increase of Monocyte and T-Lymphocyte Content in Adipose Tissue of Healthy Obese Women

**DOI:** 10.1371/journal.pone.0122872

**Published:** 2015-04-20

**Authors:** Michaela Tencerová, Jana Kračmerová, Eva Krauzová, Lucia Mališová, Zuzana Kováčová, Zuzana Wedellová, Michaela Šiklová, Vladimir Štich, Lenka Rossmeislová

**Affiliations:** 1 Franco-Czech Laboratory for Clinical Research on Obesity, Third Faculty of Medicine, Charles University in Prague, Prague 10, CZ-100 00 Czech Republic; 2 Department of Sport Medicine, Third Faculty of Medicine, Charles University in Prague, Prague, CZ-100 00 Czech Republic; 3 Second Internal Medicine Department, Vinohrady Teaching Hospital, Prague, Czech Republic; INSERM/UMR 1048, FRANCE

## Abstract

**Background/Objectives:**

Hyperglycemia represents one of possible mediators for activation of immune system and may contribute to worsening of inflammatory state associated with obesity. The aim of our study was to investigate the effect of a short-term hyperglycemia (HG) on the phenotype and relative content of immune cells in circulation and subcutaneous abdominal adipose tissue (SAAT) in obese women without metabolic complications.

**Subjects/Methods:**

Three hour HG clamp with infusion of octreotide and control investigations with infusion of octreotide or saline were performed in three groups of obese women (Group1: HG, Group 2: Octreotide, Group 3: Saline, n=10 per group). Before and at the end of the interventions, samples of SAAT and blood were obtained. The relative content of immune cells in blood and SAAT was determined by flow cytometry. Gene expression analysis of immunity-related markers in SAAT was performed by quantitative real-time PCR.

**Results:**

In blood, no changes in analysed immune cell population were observed in response to HG. In SAAT, HG induced an increase in the content of CD206 negative monocytes/macrophages (p<0.05) and T lymphocytes (both T helper and T cytotoxic lymphocytes, p<0.01). Further, HG promoted an increase of mRNA levels of immune response markers (CCL2, TLR4, TNFα) and lymphocyte markers (CD3g, CD4, CD8a, TBX21, GATA3, FoxP3) in SAAT (p<0.05 and 0.01). Under both control infusions, none of these changes were observed.

**Conclusions:**

Acute HG significantly increased the content of monocytes and lymphocytes in SAAT of healthy obese women. This result suggests that the short-term HG can modulate an immune status of AT in obese subjects.

## Introduction

Obesity represents a high risk factor for the development of various metabolic and cardiovascular diseases such as insulin resistance, type 2 diabetes, liver steatosis or atherosclerosis. The common feature of these complications is a low-grade inflammation characterized by increased circulating levels of pro-inflammatory cytokines and chemokines (e.g. IL-6, TNF-α, CCL2, CCL5) and enhanced accumulation of immune cells (macrophages, lymphocytes) in adipose tissue (AT) [1–3].

In a previous study focused on subcutaneous abdominal AT (SAAT), we found a progressive increase in the mRNA expression of macrophage markers from obese towards obese with metabolic syndrome (MS) individuals [4]. Similar findings based on a comparison of insulin-resistant with insulin-sensitive subjects were presented by other laboratories [5–7]. However, the cause of higher AT inflammation in obese subjects with metabolic syndrome compared to metabolically healthy obese remains only partly elucidated. The altered control of glycaemia on the obese background might be one factor that plays a role in the further deterioration of AT functions. Indeed, it was suggested that the deterioration of postprandial glucose control precedes long-term elevation of fasting glucose concentration [8,9]. Moreover, it was shown that fluctuations in glucose levels are more harmful than chronic hyperglycemia (HG) *per se* [10]. Detrimental effects of acute HG might be mediated through induction of oxidative stress (via production of glycosylation end product and activation of protein kinase C) and through the activation of inflammatory pathways in various cells resulting in increased secretion of pro-inflammatory cytokines [10–13]. Still, only a few reports addressed responses of cells of adaptive and innate immunity to this metabolic stimulus *in vivo* in obese individuals [14,15].

Therefore, the objective of this study was to investigate whether acute experimental HG has an impact on phenotype and relative content of monocytes/macrophages and lymphocytes in circulation and the SAAT of healthy obese women.

## Subjects and Methods

### Subjects

The co-author and the head of the Department of Sport Medicine, Vladimir Stich, MD, PhD, recruited subjects for this study among the subjects consulting at the Obesity unit of the University Hospital Kralovske Vinohrady. 30 healthy obese premenopausal women were recruited and divided into 3 groups (n = 10) matched for BMI and age (group 1- HG clamp with octreotide infusion, group 2—octreotide infusion, group 3- saline infusion study). The subjects were matched for BMI and age (range 27–32 kg/m^2^ and 40–44 years, respectively) and then they were assigned to one of the three experimental procedures without systematic randomization. All women were drug-free and without signs of metabolic syndrome [16], except for obesity. To exclude subjects with metabolic syndrome we followed NCEP-ATP III guidelines (https://www.nhlbi.nih.gov/files/docs/guidelines/atglance.pdf), i.e. only women exerting less than 3 out 5 risk factors (waist circumference > 88cm, TAG > 1.7mmol/l, HDL-cholesterol <1.3mmol/l, blood pressure > 130/85mmHg, fasting glucose ≥ 5.6mmol/l) were admitted to the study. Their body weight had been stable for 3 months prior to the examination. Participants signed a written informed consent before the study. The study was performed according to the Declaration of Helsinki and approved by the Ethical Committee of the Third Faculty of Medicine (Charles University in Prague, Czech Republic).

### Design of clinical investigation

Clinical investigation was performed before intervention in the fasting state and at the end of the 3-hour HG clamp, or octreotide, or saline infusion. Anthropometric measurements and blood processing were performed as previously reported [17,18]. Body composition was assessed using multi-frequency bioimpedance (Bodystat, Quad scan 4000, Isle of Man, British Isles). 1–2 ml of un-coagulated blood samples was used for flow cytometry analysis. SAAT was obtained by needle biopsy carried out in the abdominal region (10 cm laterally from umbilicus) under local anesthesia (1% Xylocain) as previously described [17]. Biopsies were performed 30 min before the start of the experimental infusions and within the last 15 min of infusions on the contralateral side of abdomen. 1–2 g of SAAT was used for isolation of stromal vascular fraction (SVF) cells to perform flow cytometry analyses. In a subgroup of 6 women in HG and in 9 women from the two remaining experimental groups, 0.1 g of SAAT was immediately frozen in liquid nitrogen and stored at -80°C until RNA isolation.

### Hyperglycemic clamp

A bolus injection of 0.33 g/kg glucose followed by a varying 20% glucose infusion was used to achieve steady-state plasma glucose concentrations of 15 mmol/l for 180 minutes. Continuous infusion dose was adjusted every 5 to 10 minutes according to the measured plasma glucose. 5 minutes before the priming glucose, octreotide (Sandostatin, Novartis) infusion was started in order to block the release of endogenous insulin. The initial 25 μg IV bolus administered over 1 min was followed by an infusion at the rate 30 ng/min/kg body weight. To prevent hypokalemia, 0.26 mmol/l KCl was added to the glucose infusion.

To exclude any direct effect of infusion itself or infusion of octreotide on circulating cells and on SAAT characteristics, 2 groups of subjects as control groups (n = 10 per each group) different from those participating in the HG clamp received infusion of saline or octreotide alone (i.e. in the absence of the glucose infusion) at the duration, resp. dose identical to the hyperglycemic condition.

### Isolation of SVF cells

SAAT was washed with saline, further minced and digested with type I collagenase 300 U/ml in PBS/ 2%BSA (SERVA, Heidelberg, Germany) for 1h in 37°C shaking water bath. Digested tissue was subsequently centrifuged at 200 g for 10 minutes and filtered through 100- and 40-μm sieves to isolate SVF cells.

### Flow cytometry analysis

The whole blood and freshly isolated SVF cells were used for immediate flow cytometry analyses. SVF cells were resuspended in 100 μl PBS solution containing 0.5% BSA and 2 mM EDTA and incubated with fluorescence-labeled monoclonal antibodies (FITC-conjugated antibody CD14, CD4; PE-conjugated antibody CD14, TLR2, TLR4, CD3; PerCP-conjugated antibodies CD45 and APC-conjugated antibodies CD206 and CD8) or the appropriate isotype controls (BD Bioscience, Bedford, MA) for 30 min at 4°C according to protocol of Curat et al. [19]. The whole blood samples were stained with the same set of fluorescence-labelled monoclonal antibodies as used for SVF cells (except for CD206) for 30 min at room temperature. After staining, erythrocytes were lysed by erythrocyte lysis buffer for 15 min at room temperature. Cells were washed with PBS and analysed on FACS Calibur flow cytometer with CellQuest Pro Software (BD Biosciences, NJ, USA). The number of immune cells belonging to specified populations was expressed as percentage of gated events.

### Quantitative real time PCR (RT-qPCR)

Total RNA extraction and reverse transcription (RT-PCR) were performed as previously described [17]. Before reverse transcription, genomic DNA was eliminated by DNase I (Invitrogen, Carlsbad, CA, USA). Real-time quantitative PCR (RT-qPCR) was performed using an ABI PRISM 7000 and 7500 instrument (Applied Biosystems, Foster City, CA, USA). Primers and TaqMan probes were obtained from Applied Biosystems. Results are presented as fold change values calculated by ΔΔ Ct method normalized to geometric mean of two endogenous controls (18S rRNA and GUSB).

### Determination of plasma levels of biochemical parameters

Plasma glucose and insulin were determined using the glucose-oxidase technique (Beckman Instruments, Fullerton, CA) and an Immunotech Insulin Irma kit, resp. (Immunotech, Prague, Czech Republic). Homeostasis model assessment of the insulin resistance index (HOMA-IR) was calculated as follows: ((fasting insulin in mU/l) x (fasting glucose in mmol/l) / 22.5). Circulating levels of selected bioactive molecules were measured by commercial ELISA kits: RANTES/CCL5 (Duoset, R&D Systems, Minneapolis, MN, USA) and MCP-1 (Ready-SET-Go, eBioscience, San Diego, CA, USA). Plasma levels of other parameters were determined using standard biochemical methods.

### Statistical analyses

Statistical analysis was performed using SPSS 13.0 for Windows (SPSS Inc., Chicago, IL, USA) and GraphPad Prism 6 (GraphPad Software, Inc., San Diego, California, USA). The data were log-transformed for the analyses. The effect of HG clamp or octreotide/saline infusion was tested using parametric t-test. Differences of baseline clinical data between the three groups of patients were analysed by one-way ANOVA with Tukey multiple comparison tests. To compare the effect of HG vs. control infusions, the data were analysed by two-way ANOVA with repeated measures. Data are presented as mean ± SEM. Differences at the level of p < 0.05 were considered statistically significant.

## Results

### Clinical characteristics of obese subjects

The clinical data of subjects participating in three short-term interventions are shown in Table 1. There were no significant differences in anthropometric and laboratory parameters (including fasting blood glucose, plasma insulin levels, and HOMA-IR) between HG and octreotide group of subjects. Fasting glucose levels were lower in the saline group (vs. HG group) but no other differences were found between the two groups.

**Table 1:** Characteristics of obese subjects in experimental groups.

Characteristics	Hyperglycemia (HG)	Octreotide	Saline
**N**	10	10	10
**Age (years)**	42 ± 1	42 ± 2	44 ± 2
**Weight (kg)**	86.9 ± 2.7	87.5 ± 4.4	89.0 ± 2.2
**BMI (kg/m** ^**2**^ **)**	30.8 ± 0.8	31.9 ± 1.5	31.6 ± 1.1
**Fat (kg)**	33.2 ± 1.6	34.5 ± 3.0	35.8 ± 2.1
**Waist circumference (cm)**	95.2 ± 2.6	100.8 ± 3.4	98.5 ± 2.3
**Systolic blood pressure (mm Hg)**	117. ± 4.6	124.3 ± 3.3	124.4 ± 3.5
**Diastolic blood pressure (mm Hg)**	76 ± 3.2	77.2 ± 1.5	79.5 ± 2.3
**Glucose (mmol/L)**	5.4 ± 0.1	5.2 ± 0.1	5.0 ± 0.1 ^a^
**Insulin (mU/L)**	6.5 ± 0.9	7.3 ± 0.8	6.6 ± 0.8
**C-peptide (mU/L)**	0.7 ± 0.1	0.8 ± 0.1	0.7 ± 0.1
**HOMA-IR**	1.6 ± 0.2	1.7 ± 0.2	1.4 ± 0.2
**Cholesterol (mmol/L)**	4.7 ± 0.2	4.4 ± 0.2	4.8 ± 0.3
**Triglycerides (mmol/L)**	0.9 ± 0.1	1.2 ± 0.2	1.1 ± 0.1
**HDL-C (mmol/L)**	1.3 ± 0.1	1.4 ± 0.1	1.4 ± 0.1

Data are presented as mean ± SEM

^a^ p < 0.05

hyperglycemia vs saline; BMI: body mass index; HOMA-IR: homeostasis model assessment of the insulin resistance index; HDL-C: HDL cholesterol

### Plasma insulin, C peptide and glucose levels during hyperglycemic clamp, octreotide and saline infusions

Throughout the HG clamp plasma glucose was maintained at 15 mmol/l (coefficient of variation 7.2 ± 0.7%), being approximately three times higher compared with baseline values. The addition of octreotide prevented hyperglycemia-stimulated endogenous production of insulin except at the end of the 3-hours hyperglycemia when plasma insulin and C-peptide concentrations were modestly increased (insulin 6.45 ± 0.86 mU/l at baseline vs. 10.76 ± 2.1, p < 0.05, C-peptide 0.72 ± 0.06 mU/l at baseline vs. 1.08 ± 0.18, p < 0.05).

The infusion of octreotide alone decreased plasma insulin and C-peptide below basal levels (insulin, 7.34 ± 0.84 mU/l at baseline vs. 2.42 ± 0.39 mU/l at the end of infusion, p < 0.001, C-peptide 0.76 ± 0.84 mU/l at baseline vs. 0.27 ± 0.04 mU/l at the end of infusion, p < 0.001) and this was accompanied with a slight elevation of glucose levels (baseline 5.23 ± 0.11 mmol/l, end of infusion 5.95 ± 0.27 mmol/l, p < 0.01).

Glucose, insulin and C-peptide levels remained stable during the saline infusion (data not shown).

### Monocyte/macrophage and T lymphocyte content in peripheral blood and SAAT of obese women in response to hyperglycemic clamp, octreotide and saline infusion

**Fig 1:**
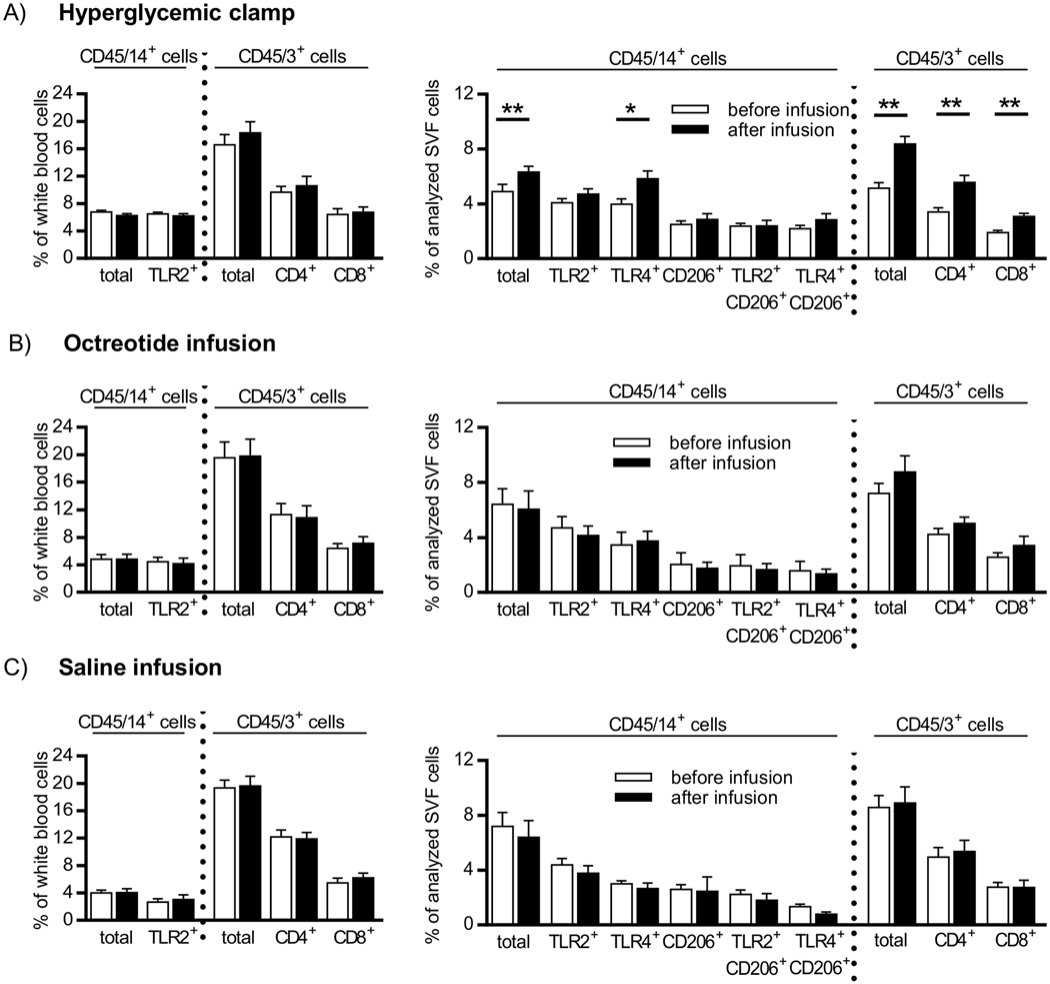
Effect of hyperglycemic clamp (A), octreotide infusion (B), and saline infusion (C) on relative content of monocyte/macrophage and T-lymphocyte populations in peripheral blood and stromal vascular fraction (SVF) of subcutaneous abdominal adipose tissue of obese women. A population of TLR4+ monocytes in blood is not shown due to a low frequency. White bars- before infusion, black bars- after infusion. Data are presented as mean ± SEM, each investigated group n = 10, *p < 0.05; ** p < 0.01: before vs after clamp.

The content of monocytes/macrophages characterized by expression of CD45+/14+ did not change in response to HG in blood but significantly increased in SAAT (Fig 1A). Similarly, no changes in relative content of monocytes/macrophages expressing Toll-like receptor (TLR) 2 and 4 were induced by HG in blood, while there was a significant HG-induced increase in relative content of CD45+/14+/TLR4+ population in SAAT (Fig 1A). These changes were independent of the content CD45+ cells with high granularity (granulocytes), in SAAT biopsy samples, because their content was not different before or at the end of the HG clamp or other experimental infusions (17.8 ± 2.3% before and 17.4 ± 1.9% after infusion, n = 30). Resident AT macrophage populations were identified by the expression of mannose receptor CD206 on CD45+/CD14+ cells (i.e. CD45+/14+/206+, CD45+/14+/206+/TLR2+ and CD45+/14+/206+/TLR4+ cells) in SAAT (Fig 1A) and they were not affected by HG.

Populations of T lymphocytes (CD45+/3+ cells; T helper subpopulation-CD45+/3+/4+; T cytotoxic subpopulation- CD45+/3+/8+) remained unchanged in response to HG in blood but significantly increased in SAAT (Fig 1A). The ratio between subpopulations of T helper and T cytotoxic lymphocytes (CD4+/CD8+) in SAAT did not change during HG clamp (data not shown). Importantly, neither octreotide nor saline infusion had a significant effect on relative content of monocyte/macrophage and T lymphocyte populations in blood and/or SAAT (Fig 1B and 1C). Of note, HG-specific increases in total T cell and T helper cell content in SAAT was confirmed by two-way ANOVA.

### SAAT mRNA levels of macrophage, lymphocyte and inflammatory markers in response to hyperglycemic clamp, octreotide and saline infusion

**Fig 2:**
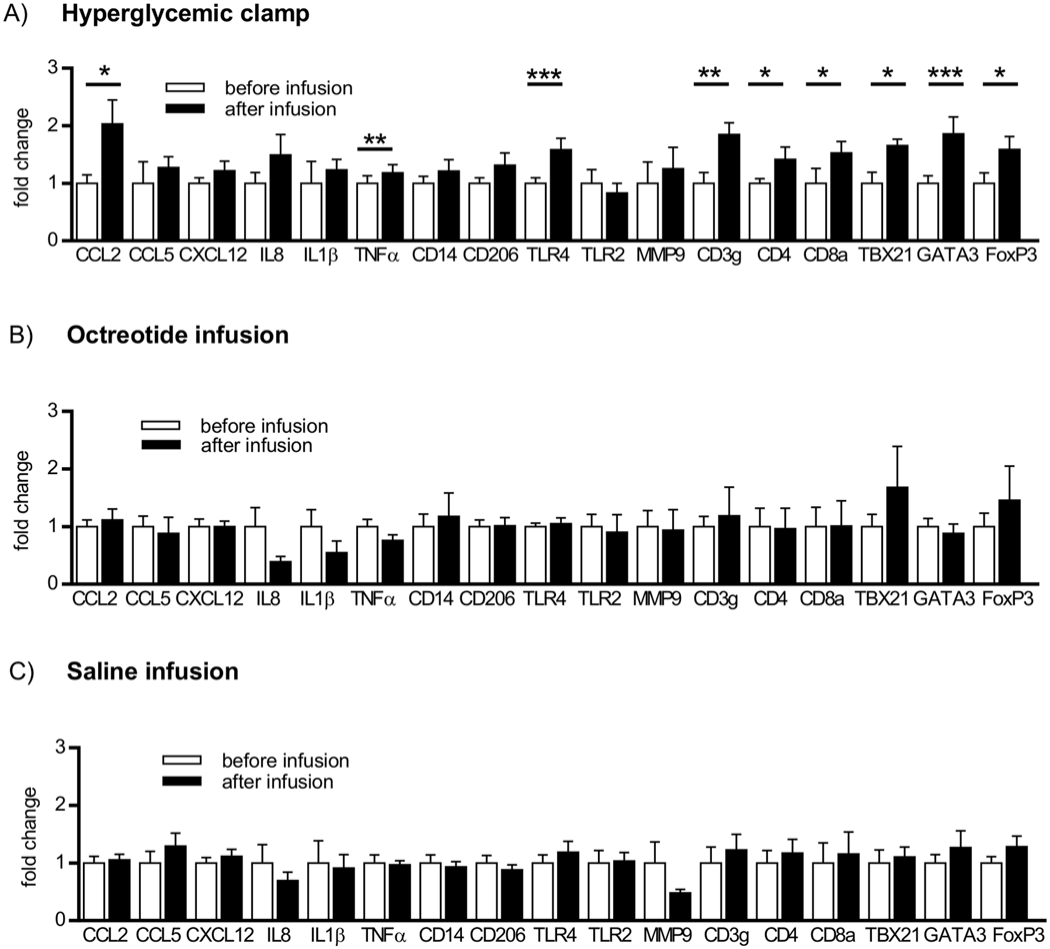
Effect of hyperglycemic clamp (A), octreotide infusion (B), and saline infusion (C) on mRNA levels of selected immunity-related genes in subcutaneous abdominal adipose tissue of obese women. White bars- before infusion, black bars- after infusion. Data are presented as mean fold change ± SEM. Relative mRNA levels are normalized to geometrical mean of 2 housekeeping genes 18S and GUSB, n = 6 (hyperglycemic clamp), n = 9 (octreotide and saline infusion), *p < 0.05; ** p < 0.01; *** p < 0.001: before vs after clamp.

To extend the results of flow cytometry, mRNA levels of chemokines/cytokines (CCL2/MCP1, CCL5/RANTES, CXCL12/SDF-1α, IL8, IL1β, TNFα), markers of macrophages (CD14, CD206), Toll like receptors (TLR2, TLR4), lymphocyte markers (CD3g, CD4, CD8a) and remodeling marker (MMP9) were analysed in SAAT. Levels of CCL2 and CCL5 chemokines were also evaluated in plasma. The mRNA levels of CCL2, TLR4, TNFα and all measured T lymphocyte markers (CD3g, CD4, CD8a) including Th1 (TBX21), Th2 (GATA3) and T regs (FoxP3) markers significantly increased in response to HG (Fig 2A) but not after octreotide or saline infusion (Fig 2B and 2C, confirmed also by two-way ANOVA).

### Plasma levels of chemokines in response to hyperglycemic clamp, octreotide and saline infusion

Circulating levels of two chemokines involved in attraction of monocytes and lymphocytes, i.e. CCL2 and CCL5, were not changed in response to either conditions (data not shown).

## Discussion

Obesity-related inflammation has been considered one of the major risk factors for the development of metabolic and cardiovascular diseases. Short-term HG represents one of the possible triggers to aberrant activation of the immune system [20]. This could contribute to the further worsening of the inflammatory state in obese subjects resulting in metabolic syndrome or type 2 diabetes. Thus, we investigated the effect of HG on immune cell phenotype and content in circulation and SAAT. The present study was carried out in healthy obese women representing an optimal model for studying the processes contributing to the deterioration of metabolic status of obese subjects.

We documented that HG induced an increase in CD45+/14+ monocyte/macrophage population in SAAT. Upon the octreotide or saline infusion, no changes in monocyte/macrophage population in SAAT were detected; therefore the above-mentioned increased numbers of monocytes/macrophage in SAAT cannot be attributed to octreotide or infusion *per se*.

Since it was shown previously that HG treatment of monocytes *in vitro* increases expression of Toll-like receptors [21] and also monocytes from patients with type 2 diabetes show a higher expression of TLR2 and TLR4 compared to healthy subjects [15], we investigated the expression of these two receptors in circulating blood cells and SAAT in obese women. While the relative content of activated monocyte/macrophage population defined as a triple positive population CD45+/14+/TLR4+ was increased in response to HG, no significant changes in CD45+/14+/TLR2+ population were observed in SAAT. Thus, the selective effect of HG on TLR4+ monocyte/macrophage population could point to a specific physiological function of this subtype of monocytes/macrophages in HG-affected SAAT. Indeed, recent findings suggest that TLR4 and TLR2 activation in macrophages results in the differential expression and secretion of pro-inflammatory cytokines [22,23]. We found increased mRNA levels of TLR4 along with TNF in the AT of obese women after HG clamp, which has been shown to be up-regulated after TLR4 but not TLR2 stimulation in macrophages [23]. This mechanism of cytokine regulation is considered to be important for the control of migration and subsequent activation of inflammatory monocytic cells. Notably, we observed that the surface expression of both TLR2 and TLR4 was detectable in the majority of monocytes present in SAAT despite low expression of TLR4 in circulating monocytes (low expression of TLR4 on circulating monocytes was also documented by Kashiwagi et al [24]). Thus, we speculate that the expression of TLR4 on monocytes could either be stimulated by the SAAT microenvironment or, alternatively, only those monocytes expressing TLR4 could reach the SAAT. However, further studies will be needed to clarify this hypothesis.

Contrary to monocyte population, a population of resident AT macrophages did not show any changes in response to HG in terms of relative content and TLRs expression (i.e. content of CD45+/14+/206+/TLR2+ and TLR4+). Therefore, it seems that SAAT microenvironment, changed by HG, activated only monocytic cells that are not fully differentiated into macrophages. Such a population of CD206- monocytic cells was described by Wentworth et al. [25] and was shown to be elevated in human obesity. It is plausible that these monocytes represent “the newest arrivals” into AT but then later can mature into CD206+ macrophages. Nevertheless, CD206 marker used to identify resident AT macrophages was previously suggested to be preferentially expressed by M2 macrophages [26], and thus it is also possible that observed increase in CD45+/14+/206- population could be attributed to M1 macrophages. This hypothesis however could not be tested as M1 macrophage marker CD40 [26] is expressed also on 60% of circulating monocytes (not shown).

The pro-inflammatory state associated with metabolic complications represents a bridging of innate and adaptive immune systems in AT physiology. Previous studies investigating the dynamics of immune cell infiltration of AT during the onset of obesity suggested that lymphocytes are the first players of immunity which infiltrate the AT [27–29]. In our study, we found an increased content of total T lymphocytes and both major subpopulations of T lymphocytes, i.e. T helper CD4+ and T cytotoxic CD8+ in SAAT of obese women in response to short-term HG. Importantly, the role of CD4+ and CD8+ T cells in modulating AT inflammation and overall metabolic status has been documented previously in both animal models and human. According to animal studies [28], CD8+ T cells direct macrophage infiltration into AT. CD4+ T cells have both anti- and pro-inflammatory roles based on their further specialization [30] and the balance between these individual CD4+ subpopulations is responsible for the control of metabolic inflammation [31]. Notably, at least two of the CD4+ subpopulations, i.e. Th1 and Th17 cells, are pro-inflammatory and their numbers are significantly elevated in AT of metabolically unhealthy obese subjects or in diet-induced obesity in mice [31,32]. Thus, we could speculate that the increase of CD4+ cells upon HG could be attributed to these two subpopulations (Th1 and Th17 cells) however this hypothesis has to be proven in further study.

In blood, short-term HG caused no alteration in relative content of immune cell populations or their phenotype, along with no change in circulating levels of chemokines, i.e. CCL2 and CCL5, involved in chemo-attraction of monocytes and lymphocytes. Thus, a short metabolic stimulus of 3-hour HG is probably insufficient to alter relative content of various leukocyte populations in circulation but it has a significant effect on immune response in SAAT of obese healthy women. In fact, relative content of immune cell populations in SAAT was analysed in the context of other cell types (i.e preadipocytes, endothelial cells) whose numbers in AT are presumably insensitive to short-term metabolic insults, which may facilitate a detection of even small changes in numbers of immune cells.

It was shown that HG modulates expression of genes related to immune response in SAAT of lean subjects [14,33]. We observed that mRNA levels of TLR4 (also expressed on adipocytes and endothelial cells [34]; [35]), CD3g, CD4 and CD8a increased in the experimental condition of HG in obese women, which nicely supports the flow cytometry results. Unlike circulating levels of CCL2, mRNA levels of CCL2 in SAAT were increased after a short-term HG. One could hypothesize that these local changes of immune response genes in the AT could affect monocyte/macrophage population. Indeed, recent paper of Amano et al. [36], suggested that CCL2 promotes proliferation of resident macrophages in AT in obesity. Likewise, other clinical studies have reported an increased expression of activation markers on monocytes and neutrophils in type 2 diabetic patients [15,37,38].

Unlike other studies analysing T cell subpopulations in mice [39], we did not analyse among T cell subtypes by flow cytometry due to the limited numbers of SVF cells derived from needle biopsy samples. However, we found the up-regulation of TBX21, GATA3 and FoxP3 mRNA levels (representing major differentiation factors of Th1, Th2 and Tregs subtypes) in SAAT after HG condition in obese women. It has been shown that Th1, Tregs are increased and Th2 subpopulation is decreased with obesity [40,41]. Based on the combination of our results from flow cytometry and mRNA analysis, one can hypothesize that HG enhanced infiltration of both pro- and anti-inflammatory T cells in order to maintain immune homeostasis in AT. However, to determine a comprehensive picture of the sequence of the immune cells activation in circulation, accumulation in AT, and their role in the induction of the AT pro-inflammatory state further analyses need to be performed. In fact, the role of immune cell infiltration in AT is not unequivocal: it still remains unknown whether it reflects the dysfunction of AT metabolism or prevents this event. The study of Duffaul et al. [42] documented that early T cells infiltration into AT has protective role since it inhibits pro-inflammatory reaction of innate cells. Similar finding by Sultan et al. [43] showed that adaptive cells alone are not responsible for the impairment of insulin sensitivity in obesity.

For characterization of particular immune cell populations in blood and SAAT, we used flow cytometry. This method enables simultaneous detection of several surface markers and provides results superior over immunohistochemistry or gene expression analysis alone. However, the flow cytometry analysis of needle biopsy-derived samples may raise concerns of a possible contamination of SAAT sample by blood cells. Similar to our previous study [18], where this possible limitation was already discussed, the content of granulocytes, i.e. CD45+ cells with high granularity, in SAAT samples, was not different before or at the end of the HG clamp or other experimental infusions used in this study. This suggests that blood contamination does not affect the outcome of the flow cytometric data in SAAT. Another possible limitation of this study was a slight increase of plasma insulin levels at the end of the HG clamp. Noteworthy, this final concentration of insulin remained within the range of normal fasting levels and was negligible when compared with the usual postprandial concentrations. In addition, the reports showing an acute effect of insulin on the circulating levels of pro-inflammatory cytokines [44,45] were based on the exposure to 4 fold higher levels of insulin than those detected in the present study. Moreover, circulating resting T lymphocytes are devoid of insulin receptor [38]. Thus even though we cannot completely rule out the possibility that the slight increase of plasma insulin may contribute to the observed effect of HG on immune cells, it seems rather unlikely.

In summary, our results show that the short-term HG induces an increase in the content of monocytes and T lymphocytes in SAAT of healthy obese women and thus suggest that the oscillations in glycaemia levels may modulate an immune status of AT in obese individuals.
